# Gastrointestinal helminth parasites of the threatened Australasian crested grebe (*Podiceps cristatus australis*, Gould 1844) in New Zealand, with descriptions of *Baruscapillaria kamanae* n. sp. (Nematoda: Trichuridae) and *Cryptocotyle micromorpha* n. sp. (Trematoda: Opisthorchiidae)

**DOI:** 10.1007/s11230-022-10022-y

**Published:** 2022-02-06

**Authors:** Bronwen Presswell, Jerusha Bennett

**Affiliations:** Department of Zoology, University of Otago, PO Box 56, Dunedin, New Zealand

## Abstract

The Australasian crested grebe *Podiceps cristatus australis*, Gould 1844 is restricted to Australia and New Zealand, where it is listed as Threatened and Nationally Vulnerable. For the first time in New Zealand, we report on the parasitic helminths infecting three individuals from Lake Wanaka, Otago, using morphological and molecular tools. Seven helminth species were found in the gastrointestinal tract: 2 nematodes (*Contracaecum ovale* and *Baruscapillaria kamanae* **n. sp.**), 4 trematodes (*Australapatemon minor, Cryptocotyle micromorpha* **n. sp.**, *Tylodelphys darbyi* and *Neopetasiger neocomensis*), and 1 cestode (*Confluaria pseudofurcifera*). Except for *T. darbyi*, all are new records for New Zealand. A change of orthography is proposed for *Neopetasiger neocomensis* and *N. pseudoneocomensis. Cryptocotyle micromorpha* **n. sp.** (Opisthorchiidae) is distinguished from similar species by its small size, wholly extracaecal vitellaria and anteriorly looped uterus. *Baruscapillaria kamanae* **n. sp.** (Trichuridae) is distinguished from other freshwater species by a combination of vulva and spicule morphology. The helminth parasites found here are mostly the same as those from the grebe in the northern hemisphere, indicating that they have been carried with the host species in its spread to Australasia. However, the parasite fauna may be depauperate due to a diminishing reservoir of intermediate hosts in that geographical migration.

## Introduction

The great crested grebe (*Podiceps cristatus* Linnaeus) (Podicipediformes) has a broad distribution in Europe, Asia and Africa, but the subspecies *P. cristatus australis* Gould occurs only in Australia and New Zealand, where it is listed as Threatened and Nationally Vulnerable under the New Zealand Threat Classification System. In addition, the species is considered taonga (“treasure”) by Maori and is fully protected. Crested grebes arrived in New Zealand over 10,000 years ago (Jensen and Snoyink, 2005), and were once widespread throughout the country. However, they are now restricted to a number of lakes in the South Island, and a survey of 2005 found the largest numbers in Lake Hayes, Otago, and Lake Heron, Canterbury (Jensen and Snoyink, 2005). In New Zealand grebes face threats due to predation by introduced mammals, loss of shore line nesting habitat and disturbances from human activities (Jensen and Snoyink, 2005). The population at Lake Wanaka, Otago, however, has increased by 300 birds in recent years (Darby, 2021), thanks to the skill and dedication of John Darby. The crested grebe is the only grebe species currently to occur in the South Island of New Zealand.

Despite its threatened status, knowledge of some aspects of Australasian crested grebe biology is still sparse. In particular, the parasite fauna of great crested grebes in New Zealand has never been examined. The benefits of discovery and identification of an animal’s parasites are well established; including data pertaining to host health, feeding habits, distribution, potential competition with other host species, and to compare with other grebe species (see Storer, 2000). Parasites can be disease causing agents too, making their study especially important for threatened host species.

Great crested grebe parasites are well documented in northern hemisphere populations. Storer (2000) listed a total of 102 helminth species (Trematoda, 46; Cestoda, 31; Acanthocephala, 2; Nematoda, 23), since which can be added a further 5 records from the literature (2 trematodes; 2 cestodes; 1 nematode) (Fain, 1956; Vasileva et al., 2003; Mutafchiev & Georgiev, 2008). Australia has geographically the closest population and is the probable origin of the New Zealand crested grebes. There are nine helminth species recorded from Australia (4 trematodes; 1 cestode; 4 nematodes), although only 5 are identified to species level and all reports are from just two studies (Johnston & Mawson, 1949; Mawson et al., 1986), so the parasite fauna from Australia is also very little known.

Due to the protected and threatened nature of crested grebes in New Zealand, it has not been possible to investigate the parasite fauna of these birds until now. However, between January 2017 and January 2020 three dead individuals were collected from Lake Wanaka, allowing the first investigation of the parasites of the New Zealand population of this subspecies. This study represents a record of the helminths found in these three grebe specimens, all of which are new records for New Zealand, and two of which are described as new species. We present brief morphological data for known species, and molecular data where required to confirm species identity, as well as descriptions of two new species.

## Materials and methods

### Morphological data

Three Australasian crested grebes (*Podiceps cristatus australis* Gould) were found dead near the marina breeding area at Lake Wanaka, Otago in January 2017, January 2018 and January 2021. Deceased birds were donated by John Darby, conservator and protector of Lake Wanaka crested grebes. A white-faced heron (*Egretta novaehollandiae* (Latham)) was also found freshly dead at a nearby site in June 2018. A black-backed gull (*Larus dominicanus* Lichtenstein) with comparable helminth infections was one of many dead birds donated by the Dunedin Wildlife Hospital from the Otago region. Birds were dissected fresh, and helminths were collected and preserved in 70% ethanol for whole-mount, 96% ethanol for genetic analyses and 4% buffered formalin for later SEM imaging.

Nematode specimens were cleared in lactophenol as temporary mounts for light microscopy. Cestode and trematode specimens were stained using acetic acid iron carmine, dehydrated in an ethanol series, cleared in clove oil and permanently mounted with Canada balsam.

Measurements were made using ImageJ software (Wayne Rasband, NIH, USA) from photographs taken on an Olympus BX51 compound microscope mounted with DP25 camera attachment. All measurements are in micrometres unless otherwise indicated, and in descriptions are given as range, followed by mean in parentheses, where numbers permit. Drawings were made with the aid of a drawing tube mounted on an Olympus compound microscope, or from photographic series.

Parasites were identified to the lowest taxonomic level possible. Taxa unfamiliar to the authors were identified using morphological keys such as Khalil et al. (1994), Gibbons (2010), Gibson et al. (2002), Bray et al. (2008), and original species descriptions. Some of the material reported in this article was described by the first author in a previous paper (i.e. *Tylodelphys darbyi*, Presswell & Blasco-Costa 2020).

Specimens of each species, when available, were chosen for scanning electron microscopy (SEM) and preserved in 4% buffered formalin. These specimens were transferred to 2.5 % gluteraldehyde in 0.1 M phosphate buffer, then post-fixed in 1% osmium tetroxide and dehydrated through a gradient series of ethanols, critical-point dried in a CPD030 BalTec critical-point dryer (BalTec AG, Balzers, Liechtenstein) using carbon dioxide, mounted on aluminium stubs, and sputter coated with gold/palladium (60:40) to a thickness of 10 nm in an Emitech K575X Peltier-cooled high-resolution sputter coater (EM Technologies, Ashford, Kent, UK). The specimens were viewed with a JEOL 6700 F field emission scanning electron microscope (JEOL Ltd., Tokyo, Japan) or Zeiss Sigma VP variable-pressure scanning electron microscope (Carl Zeiss Inc., Oberkocken, Germany) at the Otago Centre for Electron Microscopy (OCEM, University of Otago, New Zealand). Voucher specimens of new species were deposited in Te Papa Museum, Wellington, New Zealand, under accession numbers W.003609–3614.

### Molecular data and analyses

Specimens of each species were chosen for DNA sequencing. Genomic DNA was extracted using the DNeasy® Blood & Tissue Kit (Qiagen, Hilden, Germany) according to the manufacturer’s protocol. For cestodes and trematodes a partial fragment of 28S rRNA gene was amplified using T16 and T30 primers (Harper & Saunders, 2001) and conditions of 94°C for 5min, 38 cycles of 94°C for 30sec, 45°C for 30 sec and 72°C for 2min, and 72°C for 7min. Additionally, *cox1* was amplified when possible, using primers JB3 (Bowles et al., 1992) and trem.cox.rrnl (Králová-Hromadová et al., 2001) and conditions of 95°C for 2min, 40 cycles of 95°C for 30sec, 48°C for 40sec and 72°C for 1min, and 72°C for 10min. For nematodes, either the 28S, 18S or ITS1 genes were targeted using 28SF and 28SR (Nadler & Hudspeth, 2000), Nem18SF and Nem18SR (Wood et al., 2013) and SS1 and NC13R (Shamsi et al., 2008) primers respectively. The 28S PCR conditions consisted of 94°C for 5min, 38 cycles of 94°C for 30sec, 52°C for 30sec and 72°C for 1min, and 72°C for 10min. The 18S and ITS PCR conditions followed Wood et al. (2013) and Shamsi et al. (2008). All PCR products were cleaned using EXOSAP-TMTM Express PCR Product Cleanup Reagent (USB Corporation, Cleveland, OH, USA), following manufacturer’s instructions. Sanger sequencing by capillary electrophoresis was performed by the Genetic Analysis Service, Department of Anatomy, University of Otago (Dunedin, New Zealand).

Sequences were used in BLASTn searches on GenBank® to establish a preliminary identification. Sequences were imported into Geneious Prime®v1.2, trimmed using the trim function with default parameters, and manually edited for incorrect or ambiguous base calls. A contiguous sequence was assembled for each sequence, alignments were created separately for each species sequenced, and genetic divergences were calculated in MEGA v.10 comparing between closely related species to confirm identity. Newly generated sequences are listed in Table 1, with their associated GenBank accession numbers.

**Table 1:** GenBank Accession numbers of newly generated sequences of helminths from Australasian crested grebe and white-faced heron in brackets in Otago, New Zealand.

Parasite Species	GenBank Accession			
	18S	28S	ITS1	*cox1*
Nematoda				
*Contracaecum ovale*	OL470519 (OL470521)	OL470528	OL470520	
*Baruscapillaria kamanae* **n. sp.**		OL470526		
Trematoda				
*Tylodelphys darbyi*		OL470527		OL504982
*Cryptocotyle micromorpha* **n. sp.**		OL470523		OL504983
*Neopetasiger neocomensis*		OL470525		
Cestoda				
* Confluaria psudofurcifera*		OL470524		

## Results

In total, 7 parasite species were recovered from three grebe specimens: 2 nematodes, 4 trematodes and 1 cestode. All three grebe individuals were infected with at least one species of parasite. Except for the capillarid nematode, and trematode *Australapatemon minor*, each parasite species was found in all of the grebe specimens. The parasite with the highest mean intensity was a hitherto undescribed species of *Cryptocotyle,* found in many hundreds of individuals per host (infection data are summarised in Table 2).

**Table 2:** Prevalence and intensity data of helminths found in Australasian crested grebes (n=3), and heron (n=1) from Lake Wanaka, Otago.

	Australasian crested grebe		White-faced heron
	prevalence (no. birds)	Intensity range	Intensity
Nematoda			
*Contracaecum ovale*	3	24–122	245
*Baruscapillaria kamanae* **n. sp.**	2	1–13	1
Trematoda			
*Australapatemon minor*	1	3	–
*Tylodelphys darbyi*	3	2–22	4
*Cryptocotyle micromorpha* **n. sp.**	3	700–1000s	150
*Neopetasiger neocomensis*	3	40–145	800
Cestoda			
*Confluaria pseudofurcifera*	3	1–40+	–

**NEMATODA Cobb, 1932**

**Ascaridida Skrjabin et Schulz, 1940**

**Ascaridoidea Baird, 1853**

**Anisakidae Railliet & Henry, 1912**

***Contracaecum*** **Railliet & Henry, 1912**

**Type species**: *Ascaris spiculigera* Rudolphi, 1809

**Fig. 1:**
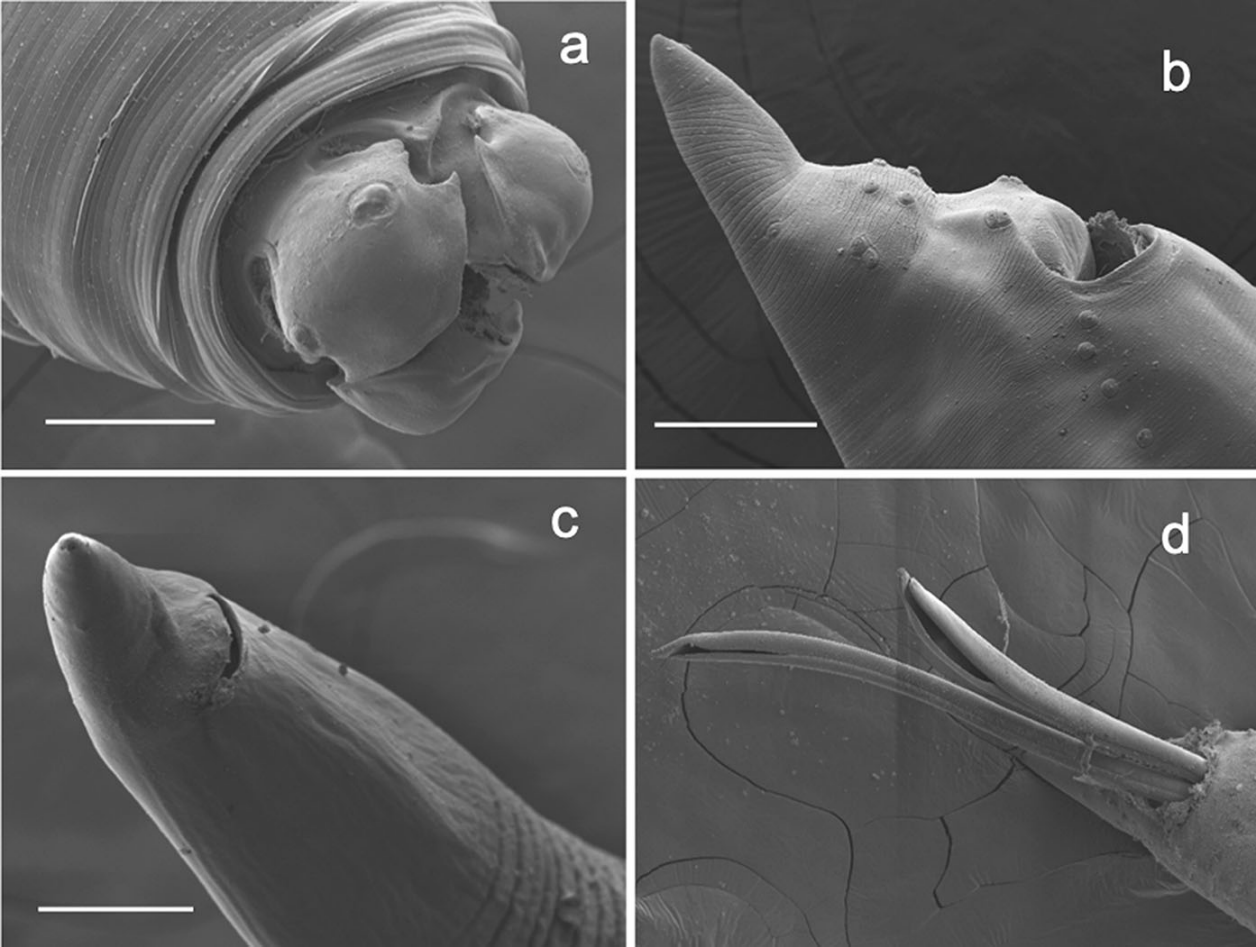
Scanning electron micrographs of nematode *Contracaecum ovale* from crested grebe, Lake Wanaka. (a) anterior end showing lips and reduced interlabia, (b) male posterior end showing caudal papillae, (c) female posterior end, (d) male posterior end showing spicules. Scale a, c, d = 100µm; b = 50µm

***Contracaecum ovale* (Linstow, 1907) Baylis, 1920.** Fig. 1a–d

Synonyms*: Ascaris ovalis* Linstow, 1907; *C. nehli* Karokhin, 1949; *C. podicipitis* Johnston & Mawson, 1949; *C. ruficolle* Vuylsteke, 1953; *C. spasskii* Mozgovoi, 1959.

All three grebes, and the single white-faced heron, were infected with specimens of *Contracaecum* Railliet & Henry, 1912, identifiable by their prominent lips without dentigerous ridges, presence of interlabia, intestinal caecum, and reduced ventriculus with solid posterior appendix. The worms (numbering 24, 53 and 122 in the three birds respectively) were mainly concentrated in the stomach, with some in the upper intestine. Infections included L4 larvae and adults, with a large size range from 11mm to 28mm. Males were smaller than females, and metrics fell within with the ranges given by Galeano & Tanzola (2012) and other references for *Contracaecum ovale*, including those of synonymous species (see below). *Contracaecum ovale* was described (as *Ascaris ovalis*) from specimens recovered from *Podiceps cristatus* in Germany. Characteristic features are indicated in Fig. 1a-d and a brief description follows.

*Males* (n = 5, plus 2 part specimens, Fig. 1b and d): body length 13.3—18.2mm; maximum body width 0.47–0.64mm; distance from anterior end to nerve ring 0.32–0.40mm (n = 4); oesophagus total length 2.4–3.4mm; intestinal caecum length 1.6–1.9mm (n = 3); spicules equal or slightly subequal, length 1.04–2.38 mm (n = 7 pairs); tail length 0.22–0.30 mm. Precloacal papillae 27–30 pairs (n = 2). Postcloacal papillae five pairs of variable placement (most common arrangement shown in Fig. 1b): first pair double, two subventral pairs, two sublateral pairs; one pair lateral phasmids level with slight constriction near tail tip.

*Female* (n = 8, Fig. 1c): body length 10.6–27.6mm; maximum body width 0.46–1.06mm; distance from anterior end to nerve ring 0.79–1.21mm (n = 2); oesophagus total length 2.8–5.6mm (n = 6); intestinal caecum length 1.9–3.5mm (n = 4); vulva in anterior half of the body, 29–34% body length; postanal length 0.34–0.62mm; eggs 60–68 x 46–56.

**Remarks**

Although the speciose genus *Contracaecum* has a confused and somewhat unreliable taxonomic history, species found mainly in grebes have distinctive characters that set them apart from other, mainly marine, species (see Discussion). Our specimens were therefore compared against all other species found in grebes, most of which we recognise as synonyms of *C. ovale*.

DNA sequences were obtained for the 18S and ITS1 markers. Closest BLASTn hits gave a larval *Contracaecum* species from a freshwater fish in India for 18S (99.63% similarity [MG696304]). It seems highly likely that the Indian specimens belong to the freshwater ‘grebe’ group of species (see Discussion). ITS1 produced a BLASTn result that included many available *Contracaecum* sequences but no other sequences were available for known ‘grebe’ species, leaving molecular identification and exploration of the relationships of the ‘grebe’ species in abeyance for the time being. The sequence of 18S was identical to those of worms from the white-faced heron.

**Enoplida Filipjev, 1929**

**Trichinelloidea Ward, 1907**

**Trichuridae Ransom, 1911**

***Baruscapillaria*** **Moravec, 1982**

**Type species:** *Capillaria obsignata* Madsen, 1945 by original designation.

**Fig. 2:**
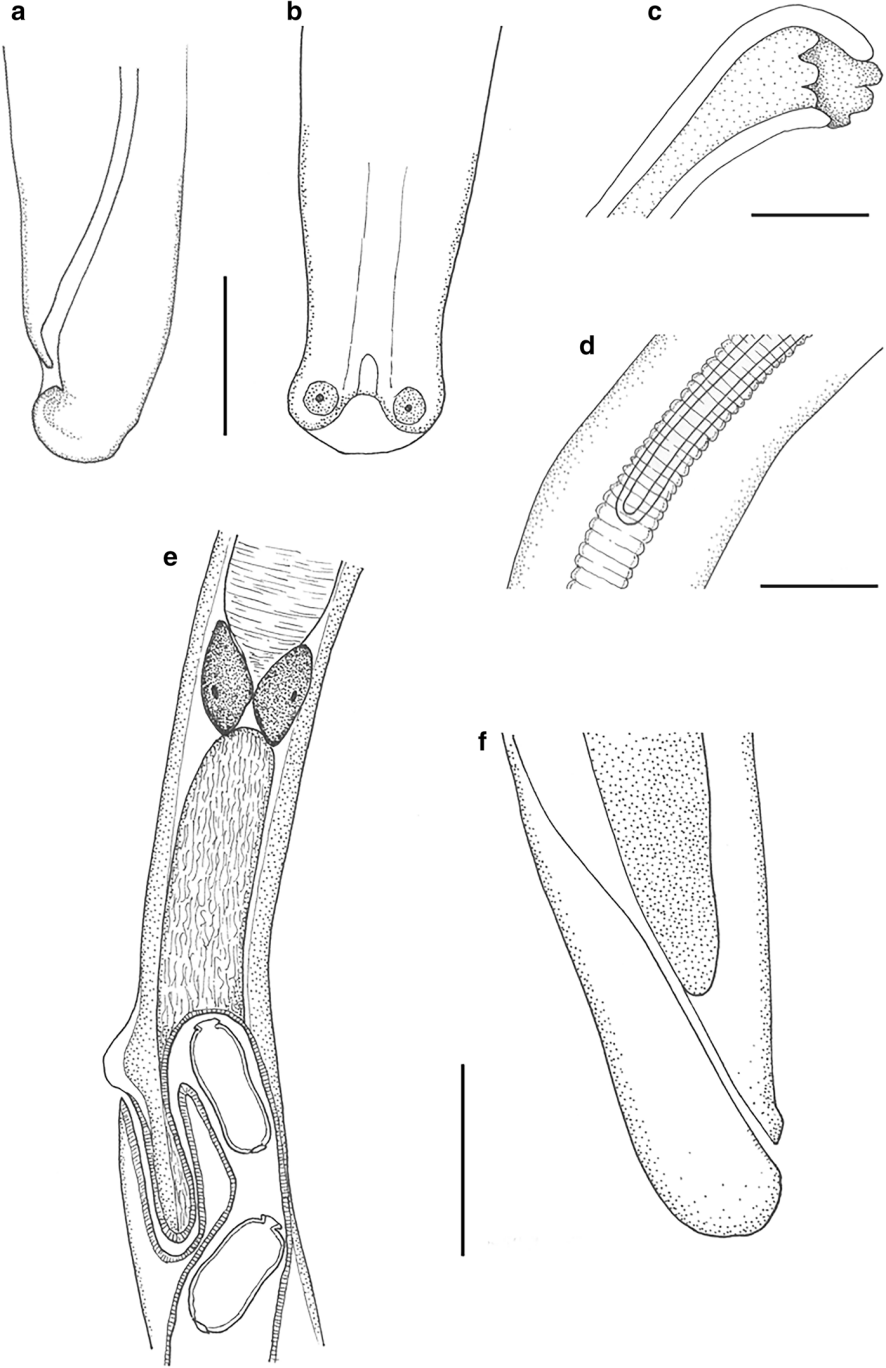
*Baruscapillaria kamanae* **n. sp.** from crested grebe, Lake Wanaka. (a) male posterior lateral view, (b) male posterior ventral view, (c) spicule proximal end, (d) spicule distal end, (e) posterior end of stichosome, showing two large glandular cells, vulva and anterior loop of vagina, (f) female posterior end. Scale a, b, c, d = 30µm; e, f = 50µm

**Fig. 3:**
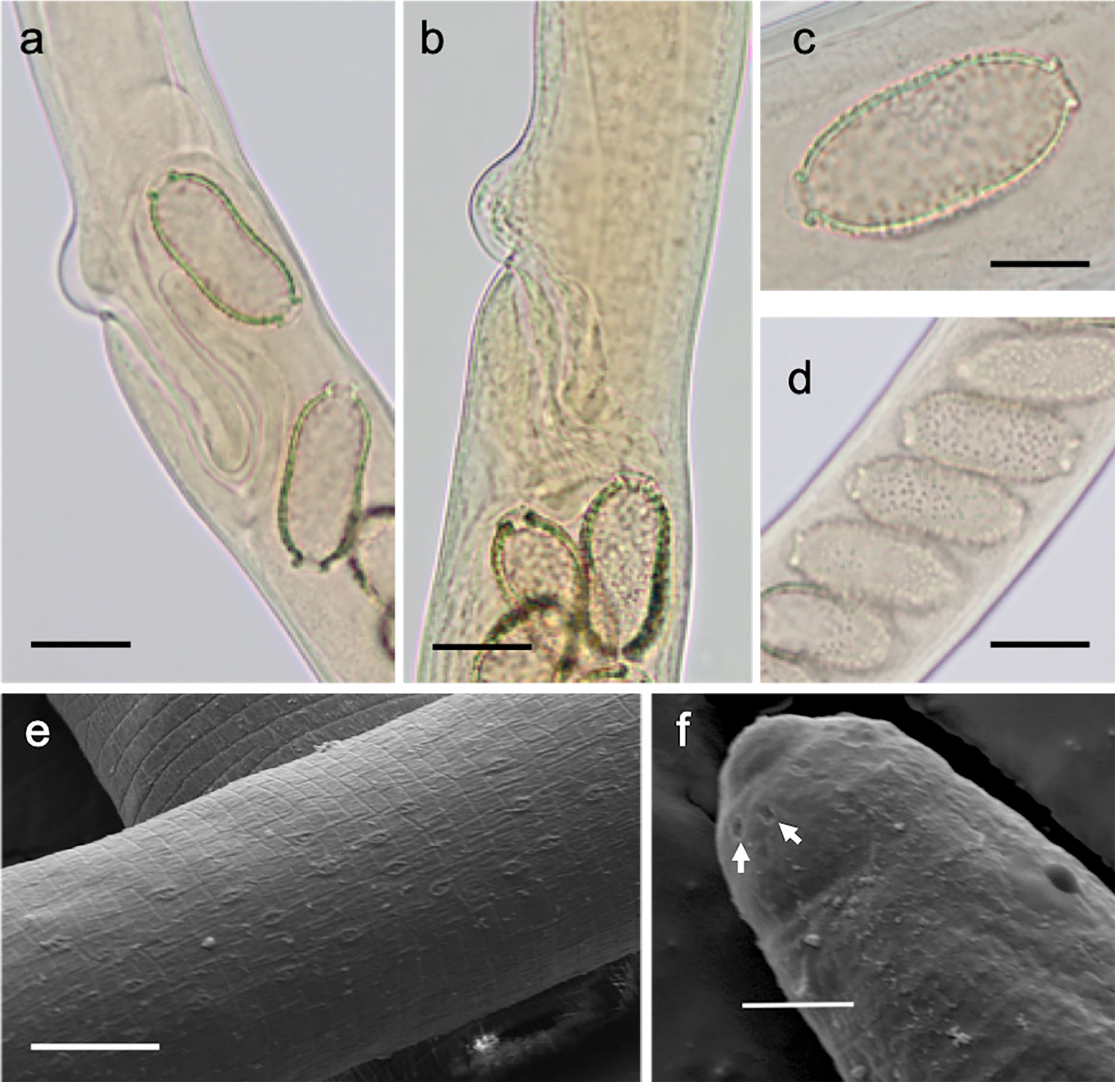
*Baruscapillaria kamanae* **n. sp.** from crested grebe, Lake Wanaka. (a) vagina with anterior loop, (b) vagina without anterior loop, (c) single egg in utero, showing polar plugs, (d) eggs in utero showing punctate surface, (e) mid-body cuticle showing bacillary band, (f) anterior end showing recessed cephalic papillae (arrows). Scale a, b, c = 20µm; c = 10µm, d = 2µm

***Baruscapillaria kamanae* n. sp*.*** Figs. 2a–f, 3a–f

A total of 16 specimens of a hair-like nematode was found in the stomachs of two grebes, of which 6 were male, 7 were female and 3 were indeterminate fragments. Two specimens of matching morphology were also found in the white-faced heron. Most of these delicate nematodes were broken, allowing some measurements, but two entire males and two entire females remained to allow body length measurements to be taken. In having a male with caudal alae absent, pseudobursa with two small, rounded lobes, a long single spicule with aspinous spicule sheath, and presence of bacillary bands, the specimens conformed to the diagnosis of genus *Baruscapillaria* (Gibbons 2010). A description is given below and comparative morphometrics are given for *Baruscapillaria kamanae* **n. sp.** and other species from freshwater bird hosts (Table 3).

**Table 3:** Measurements of *Baruscapillaria* species infecting freshwater birds.

	*kamanae* **n. sp.**				*herodiae*		*mergi* ^a^		*podicipitis* ^a^		*ryjikovi* ^a^	
Authority					(Boyd, 1966) Moravec 1982		(Madsen, 1945) Baruš & Sergejeva, 1990		(Yamaguti, 1941) Moravec 1982		(Borgarenko & Daiya, 1972) Moravec 1982	
Hosts	*Podiceps cristatus australis*				*Ardea* spp.		Gaviiformes, Podicipediformes, Ciconiiformes, Anseriformes		*Podiceps cristatus, P. auritus, P. nigricollis, P. ruficollis*.; Anseriformes; Lariformes		*Podiceps nigricollis, P. griseigena, P. ruficollis*	
Locality	NZ				USA		Europe; Russia; USA		E. Europe; East Asia, Japan		Europe; Russia Asia	
	M	n	F	n	M	F	M	F	M	F	M	F
Body length (mm) [BL]	15.3-15.8	2	23.9-24.4	2	8.3	15.3	6.8-10.5	13.7-16.6	6.3-9.0	6.8-10.1	18.4-24.2	17.9-32.3
Body width	45-55	3	76-86	3	32-40	39	48-57	55-68	44-55	44-69	50-60	49-65
Body length/width	289-363	2	278-318	2	207*	392*	142-184*	244-249*	143-163*	148-154*	368-403*	365-496*
Oesophagus length (mm)	3.9	2	7.5-10.3	2	3.7	6.7	3.6-4.4	5.3-6.5	3.7-4.3	3.6-5.2	10.9-13.0	7.6-11.3
BL/Oesophagus length	2.6	1	2.3-3.3	2	2.2*	2.3*	1.9-2.4*	2.6*	1.7-2.0	1.9	1.9-2.0	2.7
End stichosome to vulva	-		107-149	3	-	30	-	short	-	60^b^	-	150-170
Vulvar appendange	--		small swelling		-	NO	-	cup-like swelling	-	NO	-	NO
Egg length	-		45-53	20	-	46-52	-	48-53	-	42-48	-	40-53
Egg width	-		20-24	20	-	15-26	-	23-28	-	16-22	-	20-29
Spicule length	1486-1495	2	-		1250	-	1100-1250	-	630-730	-	2200-2300	-
Spicule proximal end	25-27	3	-		NG	-	17-20	-	11-16	-	23-26	-
Spicule length/BL %	9.35	1	-		15.06*	-	11.9-16.2*	-	8.1-10.0*	-	14.2-17.6	-

^*^Measurements calculated from figures in original source. ^a^Measurements taken from Baruš et al. (1978) as original description is unavailable or gives limited data. ^b^Data from Yamaguti’s (1941) original description. NG, Not Given in original source.

**Description**

*General*

Body filiform; very long and thin, tapering to anterior end, widest along length of intestine. Sexually dimorphic; males smaller than females. Lateral bacillary bands present but very indistinct, number and extent not clear. Head end narrow, rounded; oral aperture terminal, slit-like, oriented dorso-ventrally. Cephalic papillae arranged around mouth in two circles, each with six recessed papillae (Fig. 3f) Muscular oesophagus long; nerve-ring indistinct. Stichosome formed by single row of stichocytes; stichocytes long, subdivided into numerous annuli. Two large glandular cells present at junction of stichosome and intestine (Fig. 2e).

*Male* (2 entire and 4 part specimens). Body length (BL) 15.2-15.8mm, maximum width 51-55. Width of lateral bacillary band 25 (Fig. 3e). Entire oesophagus length 8.4 mm (53% of BL); junction of muscular oesophagus and stichostome not discernible, number of stichocytes not determined. Spicule well sclerotised, smooth, length 1.48-1.49 mm; proximal end expanded (23 wide) with rough protruberances, distal end rounded (9 wide) (Fig. 2c and d). Spicular sheath aspinous, with strong transverse striations. Caudal end with small membranous bursa supported by two lateral lobes each bearing ventrally projecting papilla; pseudobursa width in ventral view 30, width in lateral view 23 (Fig. 2a and b).

*Female* (2 entire and 4 part specimens). Body length (BL) 23.9-24.3 mm, maximum width 78-86. Width of lateral bacillary band 25 (n=1). Oesophagus length 7.5-10.2 mm (31-43% of BL); junction of muscular oesophagus and stichostome not discernible, number of stichocytes not determined. Vulva situated posterior to end of stichosome; distance between them 107-149. Anterior lip of vulva with small, rounded elevation; vulvar appendage absent. Vagina short, muscular, 68-120 long; initially directed posteriorly, but in some specimens with secondary anteriorly directed loop reaching level with or anterior to vulval opening (Fig. 2e, 3a and b). Eggs arranged in one to two rows; straight-sided or waisted oval, walls thickness 3; fully-developed eggs 45-53 long (including polar plugs) x 20-24 wide. Mature eggs with conspicuously punctate surface and protruding polar plugs (Fig. 3c and d), punctations less pronounced and polar plugs not protruding in immature eggs. Polar plug thickness 3, width 12. Anus subterminal; tail rounded, 12-13 long (Fig. 2f).

**Taxonomic summary**

*Type host*. Australasian crested grebe *Podiceps cristatus australis* Gould (Aves, Podicipediformes, Podicipedidae).

*Other host.* White-faced heron, *Egretta novaehollandiae* (Latham) (Aves, Pelicaniformes, Ardeidae).

*Site of infection*. Intestine

*Type locality*. Mou Waho Island, Lake Wanaka, New Zealand (44.55 S, 169.08 E)

*Other locality.* Roy’s Bay, Lake Wanaka, New Zealand (44.39 S, 168.59 E)

*Intensity.* 15, 1 and 0 in three birds.

*Type specimens*. Te Papa Museum, Wellington, New Zealand : Holotype (W.003613), Allotype (W.003614)

*Genbank accession number*. OL470526 (one partial 28S sequence)

*Zoobank reference*. urn:lsid:zoobank.org:act:2A6D33AD-DB58-48D3-AA8B-B97C6A959E37

*Etymology*. The species name, a noun in the genitive case, is derived from the Maori name for the crested grebe, kāmana.

Partial sequence of 28S was obtained for a specimen from one grebe. In BLASTn searches, the sequence showed greatest similarity with *Aoncotheca paranalis* (92.76% similarity over 445bp), *Capillaria plica* (91.22%) and *Pearsonema plica* (88.85% similarity). There are no overlapping sequences for *Baruscapillaria* spp. on GenBank, so these results tell us little at this time except for confirming the familial placement. Attempts to amplify 18S were unsuccessful.

**Remarks**

There are currently 21 species of *Baruscapillaria* that appear valid in the literature (Baruš & Sergejeva, 1990). Of these, 7 species are found in mammals, 6 in terrestrial birds and 4 in marine birds. The remaining 4 species infect freshwater birds, namely *B. herodiae* (Boyd, 1966) Moravec, 1982 in *Ardea* spp., *B. mergi* (Madsen, 1945) Baruš & Sergejeva, 1990 in Gaviiformes, Podicipediformes, Ciconiiformes and Anseriformes, *B. podicipitis* (Yamaguti, 1941) Moravec, 1982 in Podicipediformes, Anseriformes and Lariformes, and *B. ryjikovi* (Borgarenko & Daiya, 1972) Moravec, 1982 in Podicipediformes.

Compared to *Baruscapillaria kamanae* **n. sp.**, *B. herodiae* is considerably smaller in body length and spicule length, has no vulval swelling and a very short distance between vulva and stichosome (Boyd, 1966). *B. mergi* also has a smaller body length, as well as a small ‘cup-like’ vulval appendage, and fine striations on the spicule (Madsen, 1945). *B. podicipitis* is notably smaller in body length and spicule length, with a very short distance between vulva and stichosome and lemon-shaped eggs (Yamaguti, 1941). *Baruscapillaria ryjikovi* is closest to *B. kamanae* **n. sp.** in size, as well as being a grebe-specific species, but it is longer than *B. kamanae* **n. sp.** with a longer spicule, and has no vulvar swelling (Baruš & Sergejeva, 1990). An unnamed species of *Baruscapillaria* was reported by Presswell and Bennett (2021) from black shag *Phalacrocorax carbo novaehollandiae* (Stephens) in Otago. Those specimens were smaller than *B. kamanae* **n. sp.** with a shorter spicule. In addition to the anterior loop of the vagina, the above characters distinguish the New Zealand grebe specimens from other known species.

**TREMATODA Rudolphi, 1808**

**Diplostomida Olson, Cribb, Tkach, Bray, and Littlewood, 2003**

**Diplostomoidea Poirier, 1886**

**Strigeidae Railliet, 1919**

***Australapatemon*** **Sudarikov, 1959**

**Type species:** *Hemistomum intermedium* Johnston, 1904

**Fig. 4:**
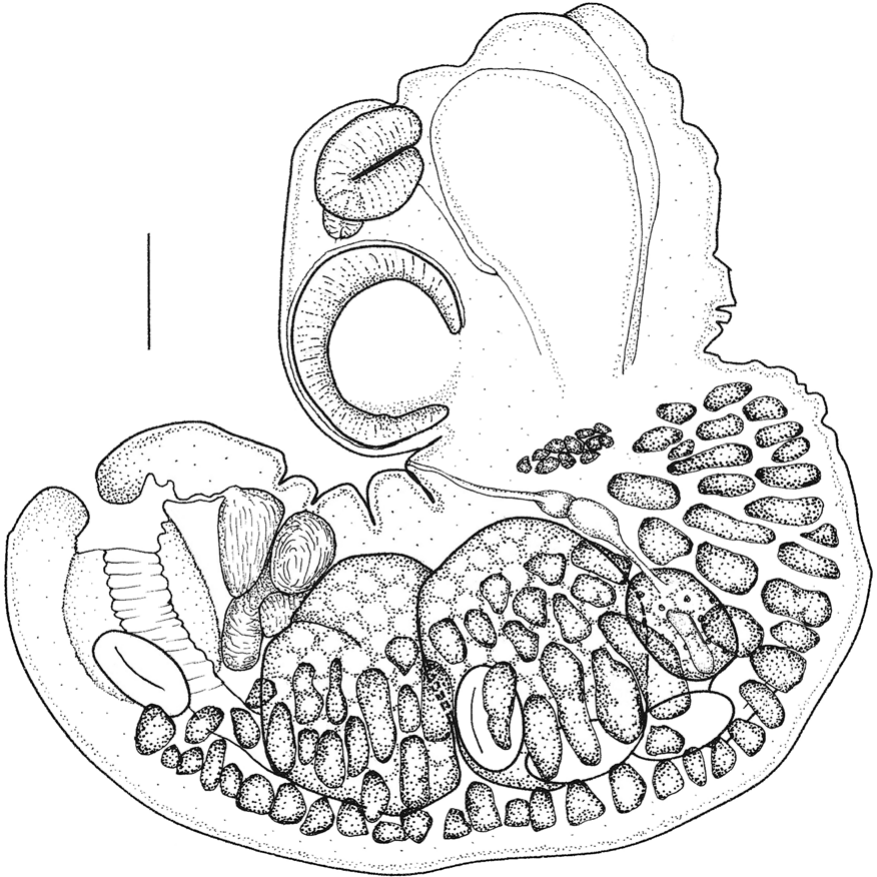
*Australapemon minor* from crested grebe, Lake Wanaka. Scale = 100µm

***Australapatemon ****minor* (Yamaguti, 1933)** Yamaguti, 1971** Fig. 4

**Synonyms:** *Apatemon minor* Yamaguti, 1933; *Apatemon gracilis minor* (Yamaguti, 1933) Dubois, 1953; *Australapatemon skrjabini* Ryzhikov, Leonov et Tzimbaluk, 1964

Three adult specimens of this species were found in the upper intestine of a single grebe.

In possessing a bipartite body, testes tandem and irregular, vitellarium confined to hindbody and genital cone large with long, rugose hermaphroditic duct, the specimens conform to the diagnosis of genus *Australapatemon* Sudarikov, 1959 as emended by Blasco-Costa et al. (2016). We include a brief description below for future comparison.

**Description**

(Based on 3 adult specimens) A small strigeid with dorsally curled body shape. Total length 1.0-1.1mm; maximum width at level of ventral sucker in forebody. Forebody 311-363 long, 327-421 wide. Hindbody 699-806 long, 329-393 wide at level of anterior testis. Ratio of forebody to hindbody length 1:2.0-2.3. Oral sucker 101-108 long, 80-97 wide. Pharynx round, 52 long, 51 wide. Ventral sucker 130-165 long, 110-143 wide. Ovary oval, 87-94 long, 115-128 wide; positioned at 22% length of hindbody. Testes bilobed; anterior testis positioned at 24-33% of hindbody, 141-195 long, 217-222 wide. Posterior testis positioned at 46-57% of hindbody, 135-141 long, 169-193 wide. Vitellaria follicular, confined to hindbody. Eggs 85-103 long, 50-54 wide. Genital cone delimited from surrounding parenchyma. Ejaculatory duct joins distal part of uterus at apex of genital cone. Hermaphroditic duct with internal rugae. Ringnapf absent.

**Remarks**

The small size of these specimens makes them comparable most closely to *A. minor*, which was described from the mallard, *Anas platyrhynchos* L. but which has also been reported from grebes (Sitko & Heneberg, 2015). The measurements of our specimens fit generally within the ranges given by Yamaguti (1933) (body length 0.8-1.2mm, forebody length 250-460, hindbody length 540-750, forebody:hindbody ratio 1:2) although the size ranges of the oral and ventral suckers and the pharynx, overlap those given for *A. minor*. In addition, the eggs of our specimens are smaller than those reported for *A. minor*. It is possible that these constitute an unreported species of *Australapatemon*, but without DNA sequence support (amplification was unsuccessful), and with few specimens, it would perhaps be premature to allocate these specimens to a new species of *Australapatemon*. We therefore tentatively attribute them to *A. minor* until more specimens allow further investigation.

**Diplostomidae Poirier, 1886**

***Tylodelphys*** **Diesing, 1850**

**Type species:** *Diplostomum clavatum* Nordmann, 1832

*Tylodelphys darbyi* Presswell & Blasco-Costa, 2019

This species was found in the upper intestine of all three grebe specimens, numbering 2, 31 and 7 respectively. Four specimens of matching morphology were also found in the white-faced heron. The species was previously described from some of the material reported herein (Presswell & Blasco-Costa, 2020) and sequence of ITS1 was provided (KU588152). Here we provide 28S and *cox*1 as additional molecular markers (Table 1). This is the first report of *T. darbyi* since its description in 2020.

**Plagiorchiida La Rue, 1957**

**Opisthorchioidea Looss, 1899**

**Opisthorchiidae Looss, 1899**

***Cryptocotyle*** **Lühe, 1899**

**Type species:** *Distoma concavum* Creplin, 1825 by original designation; *Cryptocotyle concava*, Lühe, 1899

**Fig. 5:**
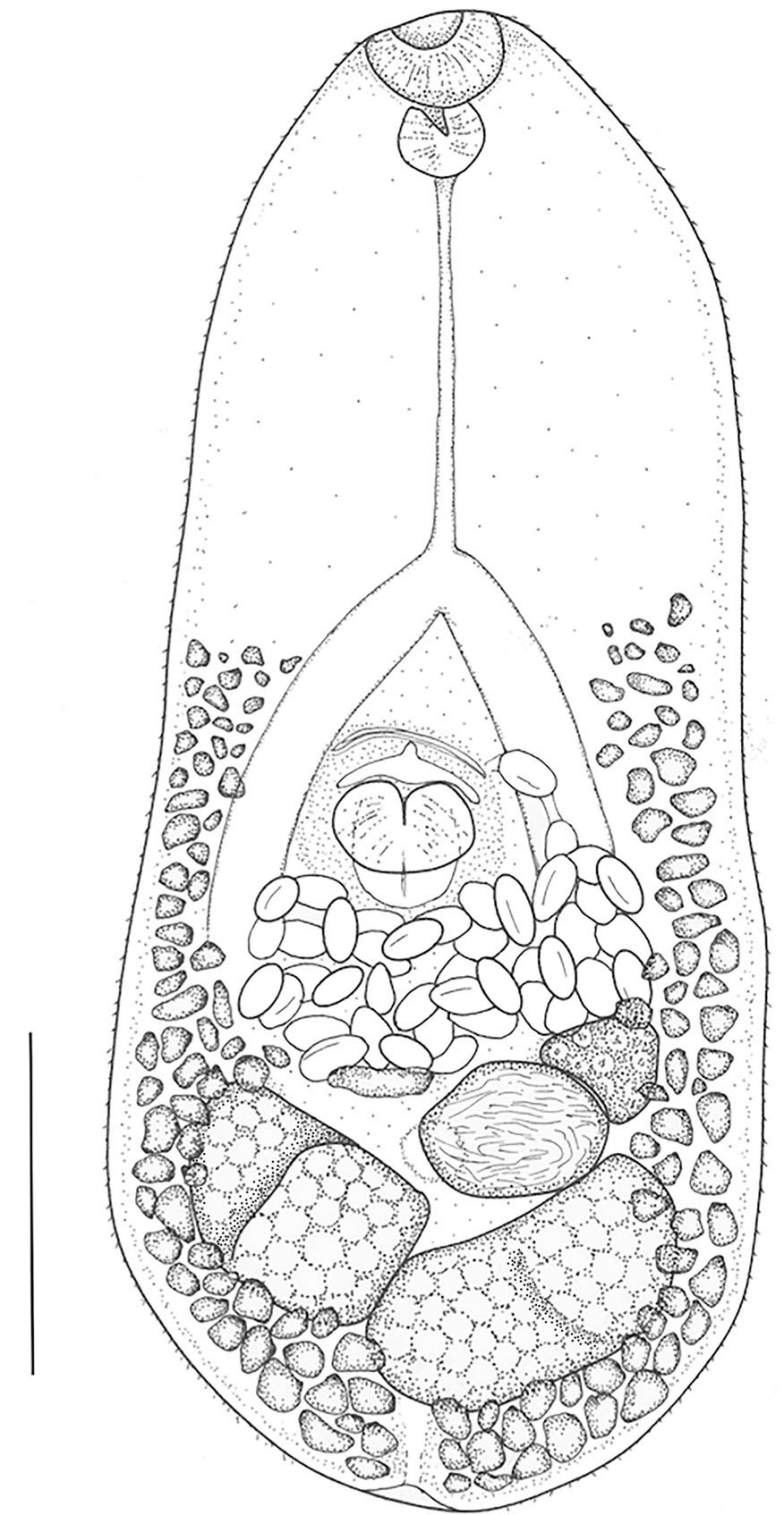
*Cryptocotyle micromorpha* **n. sp.** from crested grebe, Lake Wanaka. Scale = 100µm

**Fig. 6:**
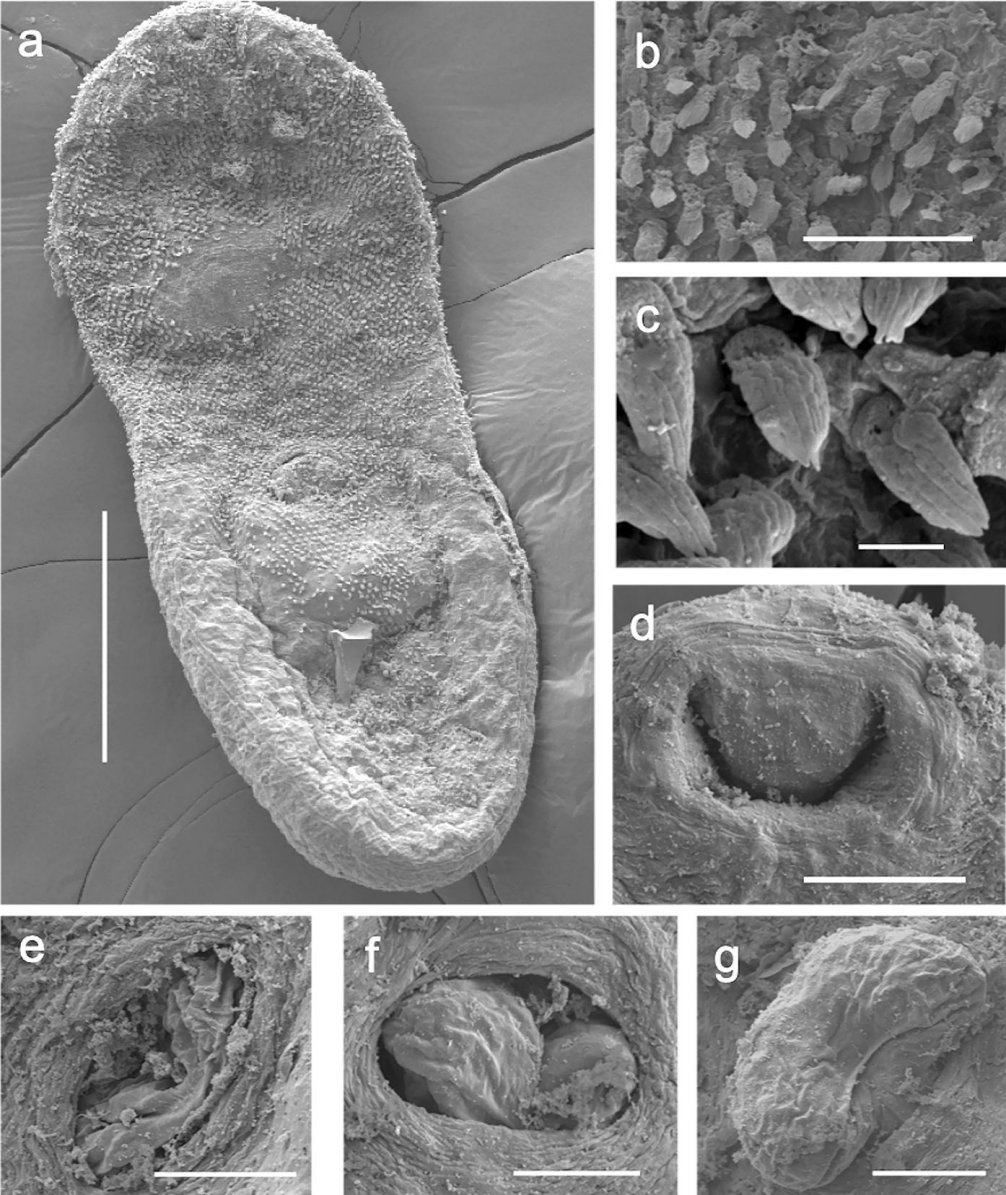
Scanning electron micrographs of trematode, *Crytocotyle micromorpha* **n. sp.**, from crested grebe, Lake Wanaka. (a) whole worm, ventral view, (b) anteroventral tegumentary scales, (c) close-up of posteroventral tegumentary scales showing 5 longitudinal lobes, (d) close up of oral sucker, (e) ventral sucker with sac retracted, (f) ventral sucker with protractile bulge semi-everted, (g) ventral sucker with protractile bulge fully everted. Scale a = 100µm; b = 10µm; c = 1µm; d, e, f, g = 20µm

***Cryptocotyle micromorpha* n. sp*.*** Fig. 5, 6a-g

Specimens of this very small trematode were found in their hundreds in all three grebe hosts, and also in the white-faced heron. In having linguiform body, tegumentary spines, ventrogenital sac with protractile bulge bearing rudimentary ventral sucker, and oblique testes they were assignable to genus *Cryptocotyle* Lühe, 1899 (Pearson, 2008). Comparison to described species of *Cryptocotyle* revealed morphological differences that indicated this was a hitherto undescribed species, and a formal description follows. Comparative morphometrics are given for *C. micromorpha* **n. sp**. and other linguiform species (Table 4).

**Table 4:** Measurements of *Cryptocotyle* species with linguiform body shape and oblique or tandem testes.

	*micromorpha* **n. sp*.***	*dominicana*	*jejuna*	*lingua*^*a*^	*thapari*	*Cryptocotyle sp.*
Authority	This study	Casalins, Arbetman, Viozzi & Flores, 2020	(Nicoll, 1907) Ransom, 1920	(Creplin 1825) Fischoeder, 1903	McIntosh, 1953	Zdzitowiecki et al. 1989
Hosts [**Type host** BOLD]	***Podiceps cristatus australis****, Egretta Novaehollandiae, Larus dominicanus*	***Larus dominicanus***	***Tringa totanus****,* gulls, terns, other birds and mammals	***Larus marinus****, L. argentatus, L. fuscus*	***Pteronura brasiliensis****, Lutra longicaudis*	***Larus dominicanus***
Habitat	Freshwater	Marine or freshwater	Marine	Marine	Freshwater	Marine
locality	New Zealand	Argentina	Europe , N. America, Asia	Europe , N. America, Asia	South America	Antarctica
Length (mm) (BL)	0.436-0.794	1.296-1.939	0.870-1300	1.5-1.7	3.1	0.720-0.900
Widest width (BW)	137-361	490-672	20% BL	400-900	790	370-450
BL/BW	2.06-3.05	2.7-2.9	2.5*	2.8*	3.9*	1.9-2.0*
BL/Forebody length	1.8-2.3	2.07*	2.18*	2.8	1.6*	1.9*
Oral sucker length	40-64	50-106	45	80	70	64-65
Oral sucker width	41-73	59-87	45	-	80	72-74
Pharynx length	22-44	42-61	38	60	80	54-61
Pharynx width	22-45	28-56	18	30	65	35-46
Oesophagus	91-139	56-154	100	28-32	175	57
Ventrogenital sac length	27-52	-	55	-	117	110
Ventrogenital sac width	34-52	-	-	-	100	100
Ovary shape	irregular, reniform, subtrianglular	irregular	oval lobed right	trilobed	irregular	lobed
Ovary length	30-67	86-168	-	80	250	70-90
Ovary width	36-82	98-375	-	180	340	140
Testes placement	Irregular oval, bilobed, oblique.	Oblique	Irregular oval indented on posterior border, oblique	Oblique	Irregular lobed, tandem, equal	Oblique
Left testis length	67-164	62-252	-	250	250	140-150
Left testis width	57-116	154-288	-	130	300	130-140
Rght testis length	66-143	-	-	-	-	170-190
Right testis width	56-128	-	-	-	-	100-120
Vitellaria	Halfway between VS & bifurcation. Extracaecal	Halfway between VS and bifurc. Intercaecal	Level with VS. Intracaecal		Between VS and bifurcation. Intracaecal	Level of bifurcation
Egg length	24-30	25-33	31-36	48	34	29-34
Egg width	13-19	14-19	16-19	22	18	12-19

^*^Measurements calculated from figures in original source. ^a^Data from Jägerskiöld, 1898.

**Description**

(Based on 26 whole-mounted, gravid worms). Body linguiform 436–794 (622) long, 137–361 (237) wide; widest at level of testes. Most specimens with posterior edges curled ventrally, forming concave body shape. Body length (BL) to width ratio 1:2.0–3.0 (2.7). Tegument with scales, 2µm long, densest on forebody. Forebody scales heptafid, more posteriorly pentafid, each lobe terminating in a point (Fig. 6b and c). Forebody 223–438 (310) long; forebody length 43–57 (50)% BL. Oral sucker subterminal, 40–64 (49) long, 41–73 (55) wide. Prepharynx very short, or absent. Pharynx spherical, 22–44 (30) long, 22–45 (32) wide. Oesophagus 91–139 (111) long. Intestinal bifurcation approximately half way between oral sucker and ventral sucker; caeca terminate close to posterior end of body. Ventrogenital sac in median third of body, 27–52 (44) long, 34–52 (44) wide; surrounded by glandular cells, containing rudimentary ventral sucker on protractile bulge of posterior sac wall, and anterior pocket. Bulge of ventrogenital sac usually protruding through sac opening to greater or lesser degree (Fig. 6e, f, g). Prominent transverse band of muscle fibres slightly anterior to ventrogenital sac opening. Margins of ventral sucker not usually discernible. Testes in posterior third of body, bilobed, oblique, long axes at right angles to each other. Left testis usually elongate oval; 67-164 (112) long, 56-128 (81) wide. Right testis subround to irregular, 66-143 (92) long, 56-128 (81) wide. Ovary usually sinistral, with irregular margins, oval to subtriangular, anterior to testes, overlapped wholly or partly by loops of uterus; 30-67 (42) long, 36-82 (57) wide. Seminal receptacle, sinistral, anterior to testes, partly overlapping ovary. Mehlis’ gland median, level with seminal receptacle. Uterus intercecal with several serpentine loops filled with up to 140 eggs in fully mature specimens, located mainly between ventrogenital sac and seminal receptacle, but with final loop anterior to ventrogenital sac; the exact path not observed but one to several eggs always found anterior to ventrogenital complex in mature worms. Vitelline follicles extend anteriorly to level midway between caecal bifurcation and ventrogenital sac, posteriorly to end of body. Eggs operculate 24–30 (28) long, 13–19 (16) wide. Excretory vesicle Y-shaped; excretory pore at posterior end of body.

**Taxonomic summary**

*Type Host.* Australasian crested grebe, *Podiceps cristatus australis* Gould (Aves, Podicipediformes, Podicipedidae).

*Other hosts.* White-faced heron, *Egretta novaehollandiae* (Latham) (Aves, Pelicaniformes, Ardeidae); Southern black-backed gull, *Larus dominicanus* Lichtenstein (Aves, Charadriiformes, Laridae).

*Site in host.* Intestine

*Type locality.* Roy’s Bay, Lake Wanaka, Otago (44°41’22”S, 169°07’49”E)

*Prevalence and intensity of infection:* approximately 600, 200 and >1000 were found in each of three grebes

*Specimens deposited*. Te Papa Museum, Wellington, New Zealand: Holotype (W.003609), Paratypes (W.003610–12).

*Genbank Accession number.* OL470523 (parital 28S gene), OL504983 (cox1 gene)

*Zoobank Registration.* urn:lsid:zoobank.org:act:57B48D73-8142-40F3-B3E5-50FD48D6DB11

*Etymology*. The species name *micromorpha*, a noun in apposition derived from the Greek "*mikros*" (= small) and "*morphi*" (= shape), refers to the small body size of this species.

Partial sequences of 28S and *cox*1 were obtained for specimens from two grebes (Table 1), both of which were identical in the two host birds. In BLASTn searches, the 28S showed greatest similarity with *Cryptocotyle lingua* (96.72% over 609bp), and the *cox*1 sequence was closest to *C. lingua* (81.95-83.96% similarity) and *C. concava* (80.54-81.95% similarity).

**Remarks**

The genus *Cryptocotyle* contains 13 species (Sokolov et al., 2016; Tatanova & Besprozvannykh 2019; Kamiya & Ohbayashi, 1975; Casalins et al., 2020), of which 9 have body shapes that are distinctly pentagonal or ovoid, with testes opposite and vitellaria extending anteriorly to the level of the caecal bifurcation: *C. badamshini* (Kurochkin, 1959) Yamaguti, 1971, *C. concava* (Creplin, 1825) Lühe, 1899, *C. cryptocotyloides* (Issaitschikov, 1923) Witenberg, 1929, *C. delamurei* (Yurakhno, 1987), *C. gyrinicola* (Dollfus & Timon-David, 1960) Sokolov, Protasova, Lebedeva, Gorlacheva & Gorlachev 2016, *C. lata* Tatanova & Besprozvannykh, 2019, *C. misgurni* (Ohyama, Okino & Ushirogawa, 2001) Sokolov, Protasova, Lebedeva, Gorlacheva & Gorlachev, 2016*, C. quinqueangularis* (Skrjabin, 1923) Witenberg, 1929, and *C. yamashitai* (Kamiya & Ohbayashi, 1975).

The remaining four species are linguiform in shape, as are the specimens reported herein. *Cryptocotyle micromorpha* **n. sp.** may be distinguished from *C. dominicana* Casalins, Arbetmam, Viozzi & Flores, 2020, *C. jejuna* (Nicoll, 1907) Ransom, 1920, *C. lingua* (Creplin, 1825) Lühe, 1899 and. *C. thapari* McIntosh, 1953 by its smaller body size and the distribution of the vitellaria which do not extend intercaecally. Additionally, in *Cryptocotyle micromorpha* **n. sp**. the metraterm loops around, and anterior to, the ventrogenital sac, a condition that does not appear to occur in any of these species.

**Echinostomatoidea Looss, 1902**

**Echinostomatidae Looss, 1902**

***Neopetasiger*** **Baschkirova, 1941**

**Type species:** *Neopetasiger skrjabini* Baschkirova, 1941

**Fig. 7:**
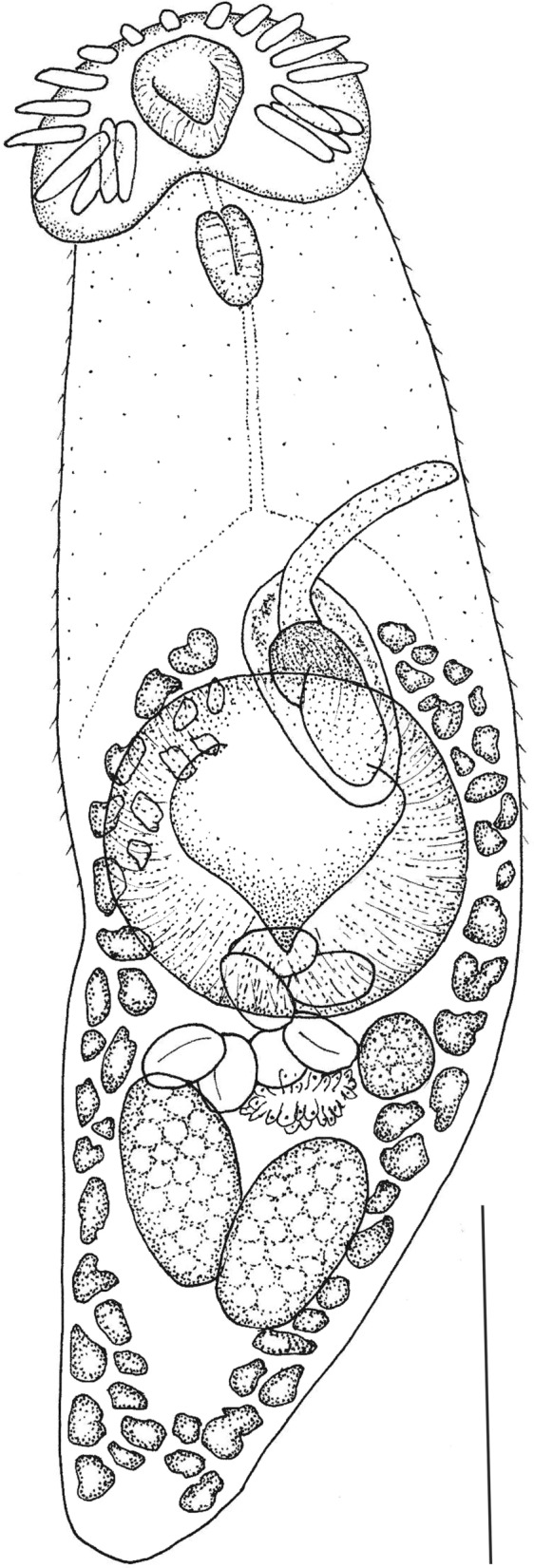
*Neopetasiger neocomensis* from crested grebe, Lake Wanaka, New Zealand. Scale = 300µm

***Neopetasiger neocomensis*** **Fuhrmann, 1927** Fig. 7

**Synonyms**: *Echinostomum pungens* Linstow, 1894 *sensu* Odhner (1910); *Petasiger longicirratus* Ku, 1938; *P. skrjabini* Bashkirova, 1941; *Petasiger megacanthus* (Kotlán, 1922) *sensu* Prudhoe (1945); *Echinostoma megacantha* (Kotlán, 1922) *sensu* Issaitschikov (1927); *P. nitidus sensu* Chen et al. (1985)

A total of 285 specimens of this small echinostomid trematode were found in the upper intestine of all three grebe hosts (145, 100 and 40 respectively), and in the white-faced heron. In having a fusiform body with a constriction in the ‘neck’ region, tegument armed with small dense scales in forebody, 19 collar spines, subspherical oral sucker, pharynx smaller than oral sucker, broadly oval cirrus sac extending to middle of ventral sucker, long tubular unarmed cirrus, post equatorial ovary and in its podicipedid host, these specimens conformed to the diagnosis of genus *Neopetasiger* Bashkirova, 1941 as emended by Tkach et al. (2016). Comparing the morphometrics and description to the 14 species listed as valid in Tkach et al. (2016) these specimens are identifiable as *N. neocomensis*, originally described from the same host, *Podiceps cristatus*, in Switzerland (Fuhrmann, 1927). We include brief measurements below.

**Description**

(Based on 11 ovigerous specimens). Body elongate, spinose to level of anterior testis ventrally, 1.18–1.46 mm long, 371–414 wide; maximum width at level of ventral sucker. Oral sucker subterminal, 73–108 , 71–104 wide. Collar with 19 spines, two groups of 4 angle spines (82–123 long), longer than lateral (52–83 long) and dorsal (65–97). Prepharynx 45–81 long. Pharynx 61–89 long, 50–63 wide. Oesophagus 124–252 long. Forebody 37–44% body length. Ventral sucker 220–312 long, 228–330 wide. Ratio of oral sucker width to ventral sucker width 1:0.29–0.41. Testes oblique, elongate oval; anterior testis 128–210 long, 78–127 wide, posterior testis 137–176 long, 85–119 wide. Cirrus sac muscular, elongate oval, 170–215 long, 72–110 wide, between intestinal bifurcation and mid-level of ventral sucker, containing bipartite seminal vesicle, and long, tubular unarmed cirrus, up to 458 long and 30 wide. Genital pore median, at level of intestinal bifurcation. Ovary oval, sinistral, pretesticular; 57–96 wide. Vitellarium in 2 lateral fields of relatively small follicles, extends from near level of genital pore to near posterior extremity; fields converge in posttesticular region.Uterus short, only 1–2 loops between anterior testis, ovary and ventral sucker. Eggs 76–83 long, 44–60 wide.

We obtained a partial 28S sequence for two specimens which were identical. In a BLASTn search these found 100% similarity (560bp) with *Petasiger* sp.1 of Selbach et al. (2014) from first intermediate hosts, *Gyraulus albus* and *Planorbis planorbis* in Czech Republic and Germany (KM191800 and KM191799).

**Remarks**

In their paper, Selbach et al. (2014) found four different cercarial or metacercarial genotypes of *Petasiger* spp., and documented morphological differences between them. The authors suggested that two of their cercarial stages could belong to *Petasiger pungens* (Linstow, 1893) and *P. neocomensis* (both species now in *Neopetasiger* – see Tkach et al. 2016)*.* In addition to this genetic match, the morphological features of our specimens, and the fact that *N. neocomensis* was described from *Podiceps cristatus* and is the only species of *Neopetasiger* recorded from this host, we believe we can confirm that *Petasiger* sp. 1 of Selbach et al. (2014) is, indeed, *N. neocomensis.*

**Orthographic note**

Storer (2000) pointed out that the name *Petasiger* is masculine and proposed Yamaguti’s (1958) use of “*neocomensis*” as opposed to “*neocomense*” as correct. We concur with this usage and add that *Neopetasiger pseudoneocomense* Bravo-Hollis, 1971 should also be changed to *N. pseudoneocomensis,* under article 34.2 of the International Code of Zoological Nomenclature.

**CESTODA Rudolphi, 1808**

**Cyclophyllidea van Beneden in Braun, 1900**

**Hymenolepididae Ariola, 1899**

***Confluaria*** **Ablasov, 1953**

**Type species:** *Confluaria spasskii* Ablasov, 1953

**Fig. 8:**
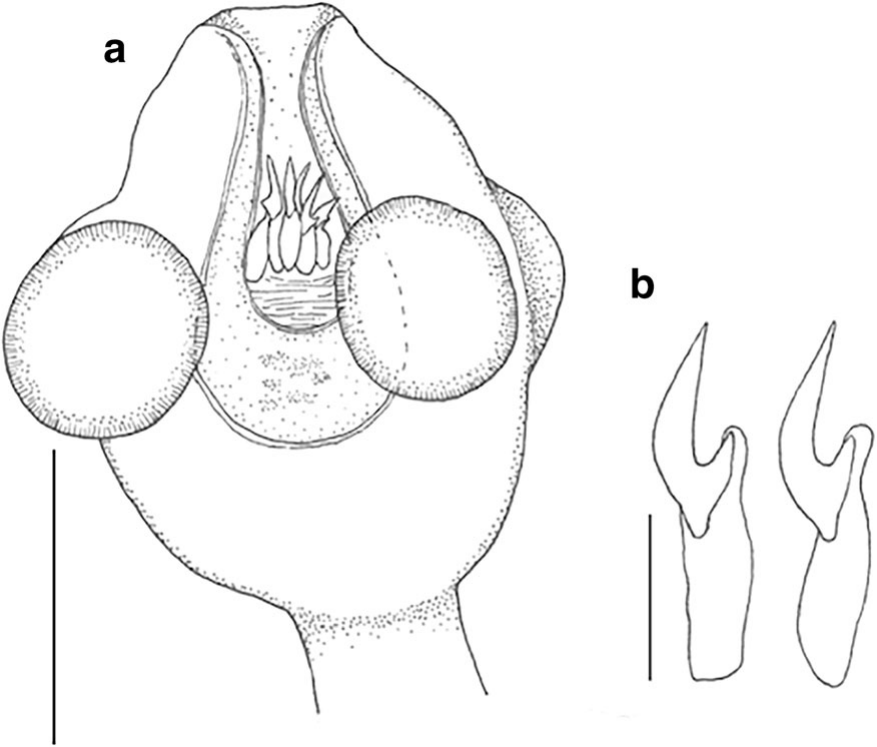
Cestode *Confluaria pseudofurcifera* from crested grebe, Lake Wanaka, New Zealand. (a) scolex, (b) 2 rostellar hooks. Scale a = 100µm; b = 20µm

***Confluaria pseudofurcifera*** **Vasileva, Georgiev & Genov, 2000.** Fig. 8

**Synonyms:** *Hymenolepis furcigera* (Krabbe, 1869) *sensu* Joyeux & Baer (1950), Jarecka (1958) and Korpaczewska (1960); *Confluaria furcifera* (Krabbe, 1869) *sensu* Galkin (1986) and Rysavy & Sitko (1995)

Specimens of this delicate cestode were found in all three grebe hosts (1 scolex; 4 scoleces and many pieces; 40 scoleces and many pieces). In having 10 rostellar hooks, aploparaksoid with large epiphyseal thickenings, invaginable rostellum, four unarmed suckers, and craspedote proglottides with unilateral genital pores these specimens could be assigned to the genus *Confluaria* Ablasov, 1953. Of the eight species currently recognised (Vasileva et al., 1999b, 2000, 2001, 2008; Bondarenko & Kontrimavichus, 2018), the hook size, and the shape and extent of the epiphyseal thickening unequivocally match those of *C. pseudofurcifera,* which was described from *Podiceps cristatus* from Bulgaria. We include a brief description below.

**Description**

(Based on 1 entire and 12 part specimens). Strobila maximum length 55mm, maximum width 784. Scolex 193-278 long, 150-181 wide, widest at suckers. Neck very long. Suckers round, unarmed, with well-developed musculature; 66-77 long, 60-72 wide. Rostellum with apical enlargement and muscular walls; width at top of rostellum 66-76. Rostellar sheath with weakly-developed musculature, length exceeds posterior margins of suckers. Rostellum with single crown of 10 hooks, each with aploparaksoid refractive particle with long straight blade, short straight handle and slightly larger guard; 19-21 long. Hooks with large epiphyseal thickening encompassing both handle and guard; hook length including epiphyseal thickening 28-37. Proglottides craspedote, much wider than long. Genital pores unilateral, central at edge of proglottis. Genital atrium deep, cylindrical. Cirrus with short, conical basal enlargement armed with rosethorn spines, seminal receptable elongate.

A partial 28S gene was sequenced for four individuals which were identical, and gave a BLAST result of assorted Hymenolepidids with a maximum similarity of 93.60%. There are currently no sequences of *Confluaria* spp. on GenBank, so no relationships can be inferred at this time.

**Remarks**

A number of works by Vasileva et al. (1999a, b, 2000, 2001, 2008) have reviewed this genus and present excellent descriptions and a key (Vasileva et al., 2000) from which the New Zealand specimens can be identified as *C. pseudofurcifera*. Only in two species does the epiphyseal thickening attach to both the handle and the guard of the rostellar hook; *C. pseudofurcifera* and *C. podicipina* (Szymanski, 1905). The epiphyseal thickening of *C. podicipina* is conspicuously more massive. Although no specimen was found with everted cirri, the base of the inverted cirrus was undoubtedly like that of *C. pseudofurcifera* with a short, thick enlargement armed with rosethorn spines.

## Discussion

We found a total of 7 helminth species infecting three crested grebes in Otago, New Zealand: 2 nematodes (*Contracaecum ovale* and *Baruscapillaria kamanae* **n. sp.**), 4 trematodes (*Australapatemon minor, Cryptocotyle micromorpha* **n. sp**., *Tylodelphys darbyi* and *Neopetasiger neocomensis*), and 1 cestode (*Confluaria pseudofurcifera*). Previous to this study there were no records of gastrointestinal cestodes or trematodes from the crested grebe in New Zealand. Apart from the two new species, and *T*. *darybi*, all the species reported herein have been recorded from *Podiceps cristatus* in other parts of the world, but all of them are new records for New Zealand. Below we discuss the taxonomic histories, known biology and life cycles of the parasites, and host specificity in the crested grebe.

Until now, no *Contracaecum* species has been reported from freshwater birds in New Zealand (McKenna, 2010). However, *C. podicipitis* (now *C. ovale –* see below) was originally described from *Podiceps cristatus australis* from Australia (Johnston & Mawson 1949). In addition to the specimens from the grebe, we also found over 100 specimens of the same species (confirmed by DNA identity) in the white-faced heron from Lake Wanaka. Although there are over 100 species in the genus *Contracaecum* (Shamsi 2019), most are found in marine environments, and there are few species found in Podicipediformes. Based on the literature, all those species reported from grebes have a distinctive head morphology, in that the interlabia are greatly reduced in size, usually reaching to about 1/3 the height of the lips, and lack any bifurcation. In addition, these species lack the conspicuous annulations of the cephalic collar, interrupted by a lateral groove seen in other, mainly marine, *Contracaecum* species. We therefore compared our specimens to only those found with these features from grebes, of which there are six nominal species: *C. nehli* Karokhin, 1949; *C. ovale* (Linstow, 1907); *C. podicipitis* Johnston & Mawson, 1949; *C*. *praestriatum* Monnig, 1923; *C. ruficolle* Vuylsteke, 1953 and *C. spasskii* Mozgovoi, 1959. The validity of all of these species has been called into question by various authors. Indeed, Macko (1961) and Hartwich (1964) regarded *C. nehli*,* C. spasskii* and *C. ruficolle* as synonyms of *C. ovale* and Baruš & Zajíček (1967) included *C. podicipitis* in its synonyms. The original description of *C. ovale* (Linstow 1907) was short on detail, and various descriptions of the species have been published since (i.e. Cram, 1927; Mozgovoi, 1953; Macko, 1961; Hartwich, 1964; Galeano & Tanzola, 2012). Those reports that gave morphometrics have greatly increased the range of measurements and interspecific variability in all features, notably the size of spicules and number of male papillae. This being the case then it seems sensible to accept the synonymisations of *C. nehli*, *C. podicipitis*, *C. ruficolle* and *C. spasskii*, which fall within the ranges given by the above authors. *Contracaecum praestriatum*, however, does appear to have some distinctive characters, not least the presence of a gubernaculum and thick lips with rounded outline, and an abrupt change in the nature of the cuticular striations at one third of the body length (Monnig, 1923). For the present the most parsimonious approach to the ‘grebe’ species is to follow previous workers (Macko, 1961; Hartwich, 1964; Baruš & Zajíček, 1967; Baruš et al., 1978) and include them all (except for *C. praestriatum*) in *C. ovale*, until such time as genetic data are available from each of the putative species to elucidate their distinctiveness or lack thereof. Further sequences from all *Contracaecum* from grebes, or other freshwater fish eating birds, should help elucidate the taxonomy and evolutionary history of this species group.

Apart from the unnamed species mentioned previously (Presswell & Bennett, 2021) the only records of *Baruscapillaria* species (reported as *Capillaria*) from New Zealand, are from terrestrial birds: *B. emberizae* (Yamaguti, 1941) in a yellowhammer *Emberiza citrinella* L. and *B. obsignata* (Madsen, 1945) in domestic fowl *Gallus domesticus* L. and rock pigeon *Columba livia* (Gmelin) (McKenna, 2010). What little is known of the life cycle of *Baruscapillaria* spp. suggests that their life histories are variable. *Baruscapillaria obsignata* has been shown to have a direct life cycle with no intermediate hosts (Moravec et al., 1987), but those species infecting grebes are believed to use oligochaetes as intermediate hosts (Storer, 2000). Although three species of *Baruscapillaria* are hosted in Podicipediformes (*B. mergi*,* B. podicipitis* and *B. ryjikovi*), only *B. podicipitis* is recorded from *Podiceps cristatus*. However, *B. podicipitis* is not host-specific, also occurring in other grebes and Anseriformes, Lariformes (Baruš et al., 1978) and Pelicaniformes (this study).

One other species of *Australapatemon* has been recorded from New Zealand; *Australapatemon niewiadomski* Blasco-Costa, Poulin & Presswell, 2016 from the mallard duck. This species is larger in all metrics than the specimens found in the grebes. The specimens of *A. minor* were found in the upper intestine. In the gizzard of the second two grebes, and in the heron, were found thousands of metacercarial cysts of the strigeid type, along with a few juvenile excysted individuals. It was clear that, despite the huge numbers of strigeid cysts found in the gizzards of these birds, only these three parasite individuals had successfully passed through into the intestine and matured. However, several questions remain concerning this phenomenon: 1) if this species is able to mature in the grebe intestine as evidenced by the three mature specimens, why did we not see 1000s of mature specimens in the intestine?, 2) could the three mature specimens belong to a different strigeid species that is able to mature in the grebe, whereas the thousands in the gizzard were able only to excyst before expiring? (unfortunately, DNA amplification did not work for the strigeid cysts, nor the three adults, so we are unable to say whether or not they are the same species), 3) *Australapatemon* spp. are known to infect leeches as their second intermediate hosts. Although it is quite feasible that grebes would eat the occasional leech, it seems unlikely that they would ingest enough to accumulate the thousands of metacercariae in the gizzard, especially considering that grebes void at least part of their gizzard contents daily as pellets (Jehl, 2017). This leaves the likelihood that the cysts belong to a species of *Apatemon*, (possibly *Apatemon* sp., “*jamiesoni*” that occurs in large numbers in bullies *Gobiomorphus cotinianus* MacDowall). This species, yet to be formally named, has been found in a mallard, and a black-backed gull *Larus dominicanus* Lichtenstein (Presswell unpubs. data). On the assumption that the cysts in the gizzard are not *Au. minor*, we have not included these cysts as part of the grebe parasite fauna, as it seems they are unable to persist in the grebe. There is no doubt that there are more strigeid species yet to be found in New Zealand (Blasco-Costa et al., 2016), so this is a puzzle that will have to await more evidence.

As well as the three grebe specimens, *Tylodelphys darbyi* was also found in a single white-faced heron from Lake Wanaka. The second intermediate host of this trematode species is the bully (*Gobiomorphus cotidianus*), in which the metacercariae inhabit the eyes. The initial report of this larval stage was from Lake Hayes, in Otago (Blasco-Costa et al., 2017). However, the species has since been found in bullies in Lakes Wanaka and Hawea (both lakes with grebe populations) and Lake Heron (Ashburton Lakes) (also with a healthy grebe population), considerably further north (Ruehle et al. in press). Despite extensive searching among the gastropod fauna of Lakes Wanaka and Hawea, the first intermediate host of this diplostomid has not yet been found.

*Cryptocotyle micromorpha* **n. sp.** is the first named species of opisthorchiid reported from a New Zealand bird; the only previous report was an unnamed species of *Metorchis* in a “wild duck” (Rind, 1974). The genus *Cryptocotyle* has traditionally been considered a member of family Heterophyidae (Pearson, 2008), but recent genetic studies convincingly place the genus into family Opisthorchiidae (Thaenkham et al., 2011, 2012; Tatonova and Besprozvannykh, 2019; Sokolov et al., 2021), and Sokolov et al. (2021) provided emended diagnoses of the family and its superfamilies to take these changes into account. However, genetic relationships within the genus *Cryptocotyle* are still relatively unknown and a larger range of taxa will be required to elucidate the interrelationships of the species, including our newly generated sequences. Caution is required when referring to records of *C. lingua* in the literature. Creplin’s (1825) description included no measurements, except for body length, and no illustrations, so identification of the species in the literature has been speculative, leading to a range of measurements that surely exceed the original species. Therefore, it is a matter of conjecture which source is used as a substitute for comparison. In this case we have used data from Jägerskiöld (1898) who confirmed identity of his specimens directly with those of Creplin. Specimens of *C. micromorpha* **n. sp.** have also been found by the authors in Southern black-backed gulls (*Larus dominicanus*), confirmed by DNA sequence identity. Morphologically comparable specimens were found too in the white-faced heron (*Egretta novaehollandiae*) from the same locality as the grebes. *Cryptocotyle* species are found in both freshwater and marine ecosystems, and gulls (Laridae) in particular are regular definitive hosts. In New Zealand, black-backed gulls (known as Kelp gulls elsewhere in the southern hemisphere) occur both at the coast and inland on freshwater lakes and rivers. One of the black-backed gulls dissected was infected only with parasites associated with freshwater, including *Cryptocotyle micromorpha* **n. sp.**

We present the first record in New Zealand of species of either *Neopetasiger* or its synonym in part, *Petasiger* Dietz, 1909. *Neopetasiger* was elevated to generic rank by Tkach et al. (2016) to include those species of *Petasiger* with 19 collar spines, predominantly parasitising grebes. The known distribution of *Neopetasiger neocomensis* includes much of central and Eastern Europe, as well as China. The history of the species includes many instances of misidentification and confusion with other species, described in detail in the revision of genus *Petasiger*, by Faltýnková et al. (2008). Their thorough and detailed study included measurements of new specimens from various different geographical and host sources, and measurements of our specimens fit convincingly within their given ranges for the species. Specimens of this species were also found in the white-faced heron from the same locality as the grebes, which constitute the first record of this species in a member of the Ardeidae.

We also present the first record in New Zealand of any species of *Confluaria*, a cestode genus that infects podicipediforms almost exclusively. In terms of numbers, cestodes constitute the dominant group in the helminth communities from grebes (Storer, 2000). Sitko and Heneberg (2015) found *Confluaria* sp. to be the most prevalent species of helminth in *P. cristatus* (80% prevalence). Specimens were found in all three of our birds, so the life cycle of this cestode obviously facilitates high prevalence, although the intermediate hosts are not known. Other species of *Confluaria* are reported to use cladocerans as intermediate hosts, although Storer (2000) suggests that it seems likely that fishes may act as paratenic or second intermediate hosts. Vasileva et al. (2000) examined specimens from several different collections and concluded that *C. pseudofurcifera* was exclusive to *P. cristatus* as they found no specimens in other grebe species.

All of the helminth parasites reported here were from the gastrointestinal tract. However, because of the rarity of dead grebe specimens, we took the opportunity to check the nasal passages of the last specimen for the possible presence of nasal schistosomes (*Trichobilharzia* spp.), the cause of swimmer’s itch, which are found as larval stages in Lake Wanaka, and in the nasal passages of New Zealand scaup *Aythya novaeseelandiae* (Norm Davis pers. comm.). The livers were also examined for this worm and any other parasites, as well as other internal organs and the eyes. No parasites were found in any of these organs in the bird that we examined.

According to Storer (2000) many helminth species are found largely or exclusively in podicipedforms, but few, if any, are known to be specific to any one species of grebe. In the northern hemisphere part of the crested grebe’s range 25 digenean species (22% of all those reported from grebes) are known only from the grebe type-host, but this figure is probably highly influenced by imprecise taxonomy and identification, rather than reflecting a true estimate of host-parasite specificity. In terms of cestodes, which show the greatest degree of specialisation to podicipediform hosts, one family, the Amabiliidae, are grebe specialists, and 15 Hymenolepididae genera are also known only from grebe hosts, with 2 species known only from their grebe type-host (Storer, 2000). Nematodes show little specificity with just 6 species recorded solely from grebes of which 2 are only known from their type-host. Storer (2000) concludes that there is no evidence for a strong pattern of specificity to single definitive host species, and that those that are reported from single grebe species more likely reflect insufficient sampling effort or geographic isolation. As several of the species found in the New Zealand grebes are the same as those found in *Podiceps cristatus* elsewhere in the world, it would seem that the NZ population has retained much of its worm fauna in its colonisation of Australasia. There are no other sympatric grebe species with which to share its parasites (the dabchick *Poliocephalus rufopectus* (Gray) and Australasian little grebe *Tachybaptus novaehollandiae* (Stephens) do not occur in the South Island), but we do find parallel infections (five out of seven helminth species) in the white-faced heron, which lives alongside the crested grebes and has a very similar diet.

Previous studies of parasite assemblages within *Podiceps cristatus* have found a more diverse helminth fauna. For instance, Sitko and Heneberg (2015) found 28 species in their birds (n = 438) from the Czech Republic. That we found only 7 species in our three birds no doubt reflects the huge difference in host sample sizes, but the lack of diversity in New Zealand may be real, and a consequence of the geographical remoteness of New Zealand and limited diversity of intermediate hosts to perpetuate infections. As the species dispersed to Australasia via the Pacific, Australia and thence to New Zealand, perhaps their parasite diversity was filtered out by an ever decreasing availability of intermediate hosts. Once in New Zealand, there were no other grebes from which to acquire more of the relatively specialist groups of parasites (Storer, 2000) that infect the podicipediforms. Recording helminth diversity is important in that it provides information regarding host health, diet, behaviour and ecology, and ecosystem health as a whole. This article provides a baseline for future research on the helminth diversity of an ecologically important bird species of Threatened status.
